# Fatty acid metabolism-related genes in bronchoalveolar lavage fluid unveil prognostic and immune infiltration in idiopathic pulmonary fibrosis

**DOI:** 10.3389/fendo.2022.1001563

**Published:** 2022-10-04

**Authors:** Yin Lyu, Chen Guo, Hao Zhang

**Affiliations:** ^1^ Thoracic Surgery Laboratory, Xuzhou Medical University, Xuzhou, China; ^2^ Department of Thoracic Surgery, Affiliated Hospital of Xuzhou Medical University, Xuzhou, China

**Keywords:** idiopathic pulmonary fibrosis, fatty acid metabolism, immune infiltration, biomarker, prognosis

## Abstract

**Background:**

Idiopathic pulmonary fibrosis (IPF) is a chronic and progressive condition with an unfavorable prognosis. A recent study has demonstrated that IPF patients exhibit characteristic alterations in the fatty acid metabolism in their lungs, suggesting an association with IPF pathogenesis. Therefore, in this study, we have explored whether the gene signature associated with fatty acid metabolism could be used as a reliable biological marker for predicting the survival of IPF patients.

**Methods:**

Data on the fatty acid metabolism-related genes (FAMRGs) were extracted from databases like Kyoto Encyclopedia of Genes and Genomes (KEGG), Hallmark, and Reactome pathway. The GSE70866 dataset with information on IPF patients was retrieved from the Gene Expression Omnibus (GEO). Next, the consensus clustering method was used to identify novel molecular subgroups. Gene Set Enrichment Analysis (GSEA) was performed to understand the mechanisms involved. The Cell-type Identification by Estimating Relative Subsets of RNA Transcripts (CIBERSORT) algorithm was used to evaluate the level of immune cell infiltration in the identified subgroups based on gene expression signatures of immune cells. Finally, the Least Absolute Shrinkage and Selection Operator (LASSO) regression and multivariate Cox regression analysis were performed to develop a prognostic risk model.

**Results:**

The gene expression signature associated with fatty acid metabolism was used to create two subgroups with significantly different prognoses. GSEA reveals that immune-related pathways were significantly altered between the two subgroups, and the two subgroups had different metabolic characteristics. High infiltration of immune cells, mainly activated NK cells, monocytes, and activated mast cells, was observed in the subgroup with a poor prognosis. A risk model based on FAMRGs had an excellent ability to predict the prognosis of IPF. The nomogram constructed using the clinical features and the risk model could accurately predict the prognosis of IPF patients.

**Conclusion:**

The fatty acid metabolism-related gene expression signature could be used as a potential biological marker for predicting clinical outcomes and the level of infiltration of immune cells. This could eventually enhance the accuracy of the treatment of IPF patients.

## Introduction

Idiopathic pulmonary fibrosis (IPF), an interstitial lung disease (ILD), is a chronic, progressive, and age-related disease. IPF has high morbidity and a poor prognosis. However, the cause of IPF is unclear (1, 2). Alveolar epithelial cells’ apoptosis, generation of pro-fibrotic factors, and stimulation of myofibroblasts and fibroblasts are involved in the pathogenesis of IPF (3–5). The Food and Drug Administration has approved two clinical drugs, nintedanib, and pirfenidone, for the treatment of IPF. However, these drugs have poor tolerability and cannot reverse the lung damage caused by IPF or reduce IPF-related death (6–8). IPF patients experience unfavorable clinical outcomes. The median survival rates are 50% and 20%, 3–5 years post IPF diagnosis, respectively, similar to lung cancer (9, 10). Consequently, IPF patients need personalized, targeted treatment, which requires developing a strategy for risk classification and the identification of prognostic genes.

Recently, substantial alterations in the metabolism of fatty acids (FAs) have been observed in IPF patients, which indicates that alterations in FA metabolism play an important role in the pathogenesis of IPF. FA metabolism pathways are complex, and disturbance in FA metabolism pathways in the lungs may result in the development of a pro-fibrotic phenotype in epithelial cells, macrophages, and myofibroblasts/fibroblasts. Sunaga et al. reported that treating cultured alveolar type (AT) 2 cells with palmitic acid induces cell apoptosis and transforming growth factor (TGF)-β1 expression in these cells (11). Mounting evidence has shown the involvement of the immune system in the progression of fibrosis and response to treatments. It has also gained extensive attention in interstitial lung disorders (12, 13). Immune cells like macrophages are predominantly distributed in the lung tissues and have an important role in the pathophysiology of pulmonary fibrosis. Macrophages can polarize into two phenotypes: M1 and M2. Once macrophages are activated, M2 macrophages release profibrotic cytokines, including TGF-β1, which stimulate the activity of fibroblasts and the deposition of extracellular matrix (14). There may be a strong association between the polarization of macrophages and FA metabolism. The M2 phenotype of the macrophages depends on the transcription factor peroxisome proliferator-activated receptor (PPAR)-γ, which plays an important role in FA metabolism as its natural ligand (15). In addition, FAs are responsible for enhancing the expression of FA receptor CD36, which induces the M2 phenotype by increasing FA uptake and self-reinforcing the profibrotic activation cycle (16). Dysfunctional epithelial cells and polarized macrophages produce numerous profibrotic cytokines, which induce the differentiation of fibroblasts into myofibroblasts. Myofibroblasts in IPF lungs produce an excessive extracellular matrix, disrupt the basement membrane, and are considered primary effector cells (17, 18). Reddy et al. have shown that nitrated FAs are PPAR-γ receptor agonists and stimulate the dedifferentiation of myofibroblasts by inhibiting the impacts of TGF-β1 (19). Furthermore, FA and their derivatives modulate CCAAT enhancer-binding protein (C/EBP) α and promote the dedifferentiation of myofibroblasts to lipofibroblasts (20). Therefore, targeting FA metabolism is an innovative approach for the treatment of IPF and is currently tested at the pre-clinical stage on pulmonary fibrosis models (21–23). However, the role of fatty acid metabolism-related genes (FAMRGs) in IPF pathogenesis is poorly understood, and the prognostic significance of these genes is yet to be elucidated.

Currently, lung biopsy is widely used for determining the molecular biomarkers for diagnosis and prognosis of IPF. However, lung biopsies are invasive, and hence their applications are limited. Prasse A et al. reported that the gene expression patterns of bronchoalveolar lavage (BAL) cells could predict mortality in IPF patients (24). Some studies showed that the levels of saturated long-chain FAs, including stearic acid, oleic acid, and palmitic acid, were low in bronchoalveolar lavage fluid (BALF) of IPF patients, in contrast, others showed that the levels of stearic and palmitic acid were high in BALF of IPF patients (25, 26). This may seem contradictory, but it gives us a clue that the changes in FA metabolism in the alveolar compartment of IPF patients could indicate disease progression.

In this study, we have analyzed FAMRGs in BAL cells to comprehensively examine the impacts of FA metabolism on the survival of IPF patients. In addition, a risk score model based on FAMRGs was developed to examine the predictive ability of FAMRGs in IPF patients. These results may offer novel insights into understanding the molecular basis of IPF. It may also provide a novel approach to the targeted therapeutic for IPF and facilitate the development of personalized therapy for IPF patients.

## Materials and methods

### Datasets and samples

For this study, gene expression profiles and clinical data of 176 IPF patients were used. Gene expression of BAL cells from the GSE70866 dataset was retrieved from Gene Expression Omnibus (GEO) database (http://www.ncbi.nlm.nih.gov/geo/). The training cohort consisted of 112 IPF patients (from Siena, Italy, and Freiburg, Germany). The RNA microarray chips from IPF patients were performed using the Agilent-028004 SurePrint G3 Human GE 8x60K Microarray. The validation cohort consisted of 64 IPF patients (from Leuven, Belgium), and the RNA microarray chips from these patients were performed using Agilent-039494 SurePrint G3 Human GE v2 8x60K Microarray. The details of the patients included in this study are presented in **Table 1**. In addition, the raw data of IPF patients from the Freiburg and Siena groups were merged into the training cohort using the R package inSilicoMerging (27). The batch effect was removed using the Empirical Bayes method (28). The Uniform Manifold Approximation and Projection (UMAP) method was used to determine if the batch effect was eliminated (29). Data on 309 FAMRGs were obtained from the databases like Kyoto Encyclopedia of Genes and Genomes (KEGG), Hallmark, and Reactome.

**Table 1:** Patients’ features in the training and validation cohorts.

Variables	Training Cohort	Validation Cohort	P-value
n	112	64	
Group, n (%)			< 0.001
Freiburg	62 (35.2%)	–	
Siena	50 (28.4%)	–	
Leuven	–	64 (36.4%)	
Sex, n (%)			0.726
Female	19 (10.8%)	13 (7.4%)	
Male	93 (52.8%)	51 (29%)	
Status, n (%)			< 0.001
Alive	36 (20.5%)	40 (22.7%)	
Dead	76 (43.2%)	24 (13.6%)	
Age, median (IQR)	69.5 (62, 76)	68.5 (63.75, 75)	0.920
Survival time (days), median (IQR)	569.5 (291, 961.25)	566.5 (346, 963.75)	0.625

IQR, Interquartile range.

### Identification of molecular subgroups

Univariate Cox regression analysis discovered 95 genes associated with the prognosis of IPF patients. Consensus clustering was performed based on the expression matrix of the 95 genes and was carried out using the R package ConsensusClusterPlus (30).

### Enrichment and immune analysis

Gene Set Enrichment Analysis (GSEA) was carried out using the same dataset to examine the differences across the clusters. Meanwhile, Cell-type Identification by Estimating Relative Subsets of RNA Transcripts (CIBERSORT) (31) analysis was used to determine the proportions of 22 human immune cell subsets in the BALF of IPF patients based on the gene expression data.

### Construction and verification of the risk model

Least Absolute Shrinkage and Selection Operator (LASSO) regression analysis was performed using R package glmnet, based on a 5-fold cross-validation in the training cohort to filter out FAMRGs associated with the survival of IPF patients. The lowest lambda value was determined as the optimal value. LASSO regression analysis identified genes related to the prognosis of IPF patients (survival time and status). These genes were used to generate a prognostic risk signature based on the regression coefficients. The following formula was used to determine the risk score of each patient within the training and validation cohorts: Riskscore = Σ Exp (mRNAi) × Coefficient (mRNAi). Patients were categorized into low- and high-risk groups based on the median value. The predictive ability of the model was assessed using the survival analysis and time-dependent receiver operating characteristic (ROC) curves.

### Statistical analysis

R package (version 4.0.2) was used to perform statistical analysis. The Kaplan-Meier survival analysis and the log-rank test were used for survival analyses. The predictive accuracy of the risk model was assessed by the time-dependent ROC using the R package survivalROC. The patients were classified based on age and gender for subgroup analysis. The student’s t-test was used to compare the two groups, and the comparisons among multiple groups were carried out using one-way ANOVA. Statistical significance was defined as p < 0.05.

## Results

### Removal of batch effects in the training cohort

**Figure 1:**
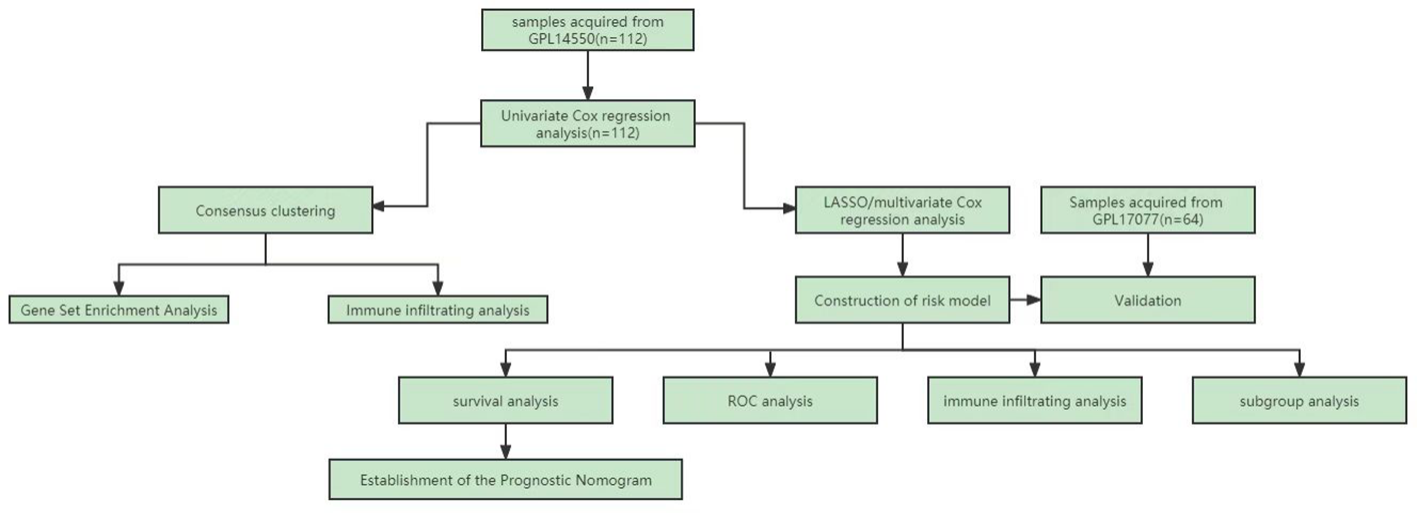
The flow chat of this study.

**Figure 2:**
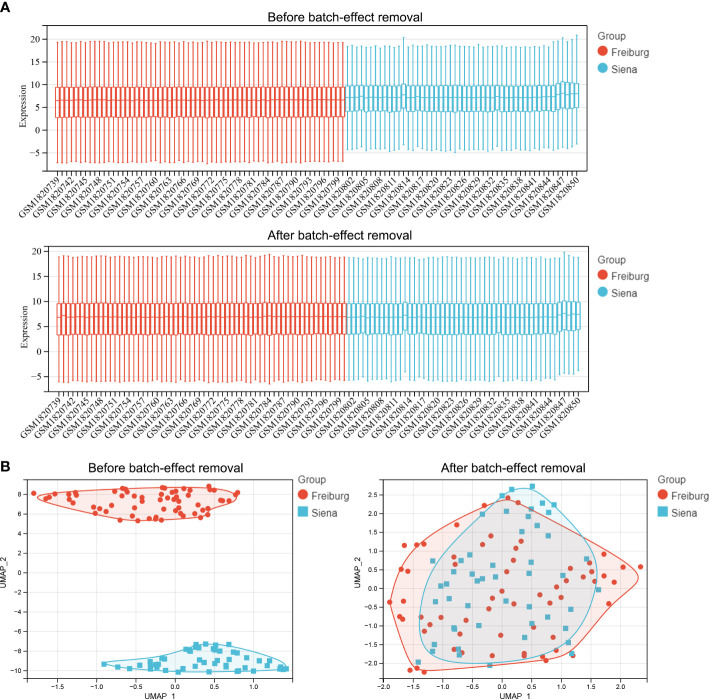
Comparison of expression data distribution and Uniform Manifold Approximation (UMAP) distribution before and after removal of the batch effect. **(A)** Expression data distribution. **(B)** UMAP distribution. The colors distinguish the Freiburg and Siena groups.

**Figure 1** shows the flowchart of the workflow of the study. The empirical Bayes method was used to eliminate batch effects between the Freiburg and Siena groups in the training cohort. The boxplot shows that the sample distribution of each group differed significantly before the elimination of the batch effect, indicating the existence of the batch effect. Once the batch effect was removed, the data distribution of each group was consistent (**Figure 2A**). UMAP results show that the samples from each group clustered together before batch effect elimination, suggesting the presence of the batch effect. Once the batch effect was eliminated, the samples from each group clustered together and intertwined, suggesting that the batch effect was eliminated (**Figure 2B**).

### Molecular subtype identification prediction based on FAMRGs

**Figure 3:**
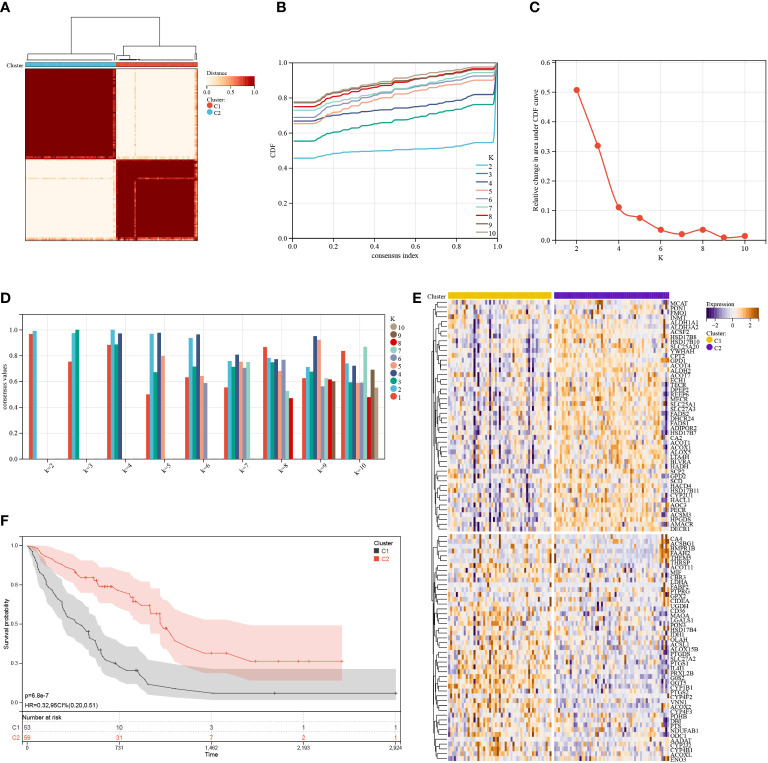
Consensus cluster. **(A–D)**: K = 2 was identified as the optimal value for consensus clustering. **(E)** Heatmap shows the expression of fatty acid metabolism-related genes in the two clusters. **(F)** Survival curve of the patients in the two clusters.

The consensus clustering method was used to classify the IPF patients from the training cohort into distinct groups based on 95 prognostic genes obtained by univariate Cox analysis (**Table S1**). K = 2 was identified as the optimum value for clustering stability (**Figures 3A** **–D**). Cluster 1 (C1) included 53 IPF patients, and cluster 2 (C2) had 59 IPF patients. The expression levels of FAMRG in the two clusters were visualized using the heatmap (**Figure 3E**), and the results revealed that C1 and C2 had significantly different gene expression patterns. Further, the patients in C2 exhibited a significantly higher (p < 0.0001) overall survival (OS) compared to patients in C1 (**Figure 3F**). These results show that IPF patients can be classified into two distinct molecular subgroups based on FAMRGs, and each subgroup had a different OS. Hence, these results indicated that it was possible to create a prediction model based on FAMRGs.

**Figure 4:**
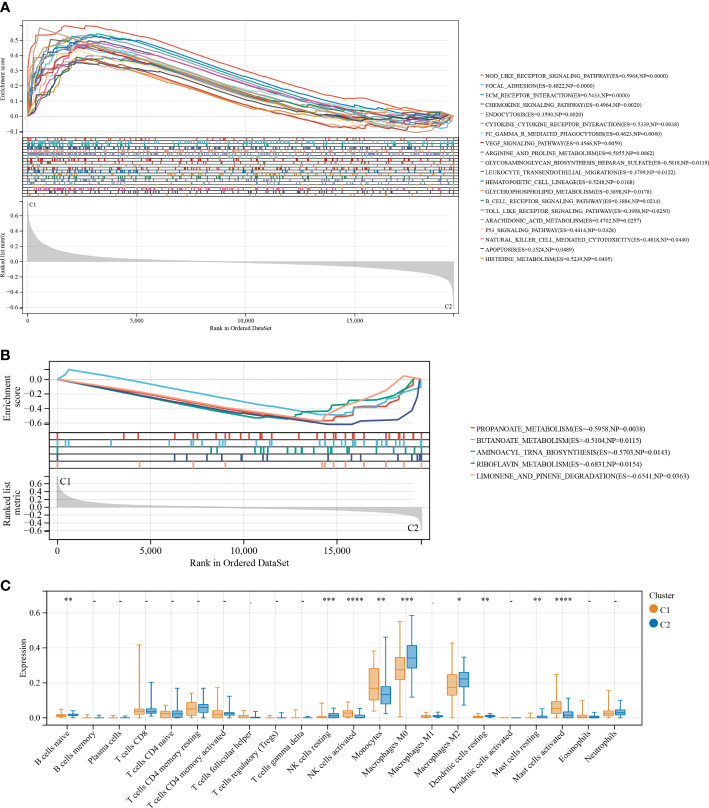
Enrichment analysis and the immune infiltration characteristics of the two identified subgroups with distinct prognoses. **(A)** GSEA for cluster 1 (C1) and cluster 2 (C2). Immune and metabolism-related pathways were significantly enriched by cluster 1. **(B)** and another five metabolism-related pathways were significantly enriched by cluster 2. **(C)** Boxplots shows differences in the infiltrating immune cells between clusters 1 and 2. *p < 0.05; **p < 0.01; ***p < 0.001; and ****p < 0.0001.

GSEA was used to elucidate the possible mechanisms responsible for the difference between C1 and C2. As shown in **Figure 4A**, immune-related pathways, including the Nod-like receptor signaling pathway, chemokine signaling pathway, leukocyte transendothelial migrations, natural killer cell-mediated cytotoxicity, B cell receptor signaling pathway, Toll-like receptor signaling pathway, and metabolism-related pathways (i.e., arginine and proline metabolism, glycosaminoglycan biosynthesis heparan sulfate, glycerophospholipid metabolism, arachidonic acid metabolism, and histidine metabolism) were significantly (p < 0.05) enriched by cluster 1, indicative of poor survival. Further, five metabolism-related pathways, including propanoate metabolism, butanoate metabolism, aminoacyl tRNA biosynthesis, riboflavin metabolism, and limonene and pinene degradation, were significantly (p < 0.05) enriched by cluster 2 and were different compared to pathways enriched by cluster 1 (**Figure 4B**). Taken together, these findings show a correlation between the FAMRGs expression and dysregulation of the immune system and changes in nutrient metabolism status. This may be linked to the poor prognosis of IPF patients.

To establish the association between clusters and the infiltration of immune cells in the BALF of IPF patients, a CIBERSORT analysis was conducted to compare the relative levels of immune cells in C1 and C2 (**Figure 4C**). High infiltration of activated natural killer (NK) cells, monocytes, and activated mast cells were observed in the BALF of IPF patients in C1. The infiltration levels of resting dendritic cells, naive B cells, resting mast cells, M0 and M2 macrophages, and resting NK cells were high in the BALF of IPF patients in C2. These results demonstrate considerable differences in the immune status of the two clusters.

### Construction of FA metabolism-related prognostic risk model

**Figure 5:**
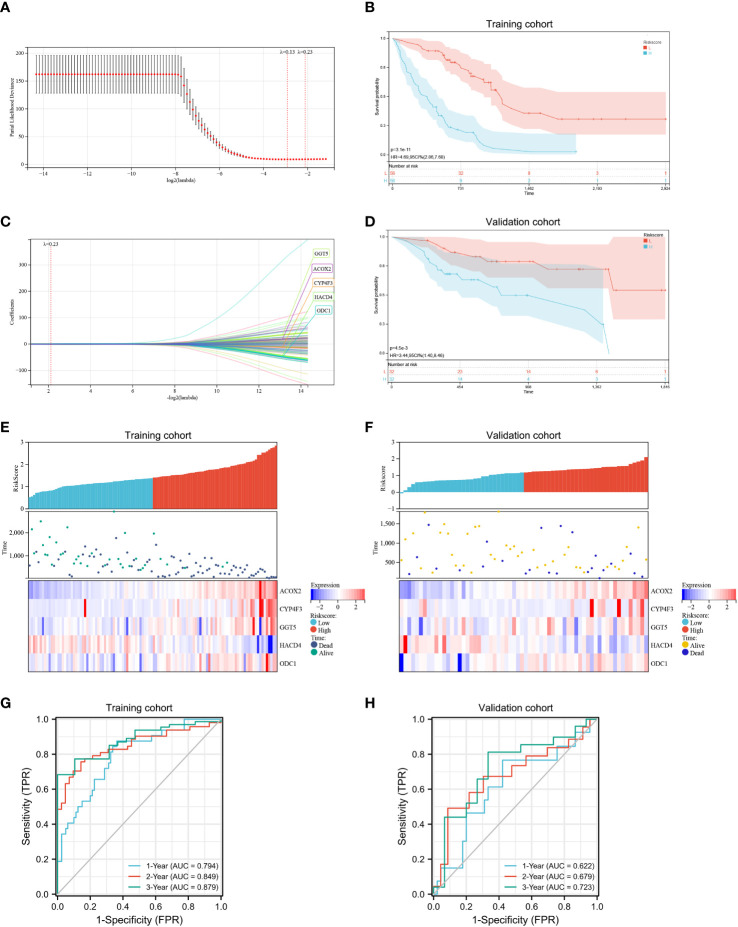
Development and assessment of a prognostic fatty acid metabolism-related signature for patients with IPF. **(A, B)** LASSO regression analysis with optimal lambda. **(C, D)** Kaplan–Meier survival analysis of patients with high- and low- Riskscore in training **(C)** and validation cohorts **(D)**, IPF patients in the high- Riskscore group had poor survival prognosis compared to patients in the low- Riskscore group. **(E, F)** Distribution of the Riskscore, survival status, and the expression of the five candidate genes in the high- and low- Riskscore groups in the training **(E)** and validation **(F)** cohorts. **(G, H)** In the training **(G)** and validation **(H)** cohorts, time-dependent receiver operating characteristic analysis showed that the Riskscore had better performance in predicting the survival of patients with IPF.

Next, a risk signature model was created to determine the contribution of FAMRGs for the accurate prognosis of IPF patients. The LASSO algorithm and the lambda value set at 0.23 were used to identify the most reliable prognostic FAMRGs (**Figure 5A**). Five genes (*GGT5, ACOX2, CYP4F3, HACD4*, and *ODC1*) were identified and used to generate the FAMRGs-associated prognostic risk model (**Figure 5B** **)**. The Kaplan-Meier survival analysis shows that each gene could independently serve as a prognostic indicator for IPF patients (**Figure S1**). The following formula was used to determine the Riskscore for each patient in the training and validation cohorts:

Riskscore = 0.0949389697248446 × expression value of *GGT5* + 0.315559675709913 × expression value of *ACOX2* + 0.00450989430661515 × expression value of *CYP4F3* − 0.284593576349762 × expression value of *HACD4* + 0.190681441039345 × expression value of *ODC1*.

The patients were classified into low- and high-risk groups based on the median score. Kaplan-Meier survival analysis (**Figures 5C** **, D**) shows that IPF patients in the high-risk group experienced significantly more rapid progression compared to IPF patients in the low-risk group in both the training (p < 0.0001) and the validation cohort (p < 0.01). Therefore, the established prognostic risk model successfully grouped the patients with IPF into low- and high-risk groups. Moreover, high expression of the four candidate genes (*GGT5, ACOX2, CYP4F3*, and *ODC1*) and low expression of a candidate gene (*HACD4*) were observed in the high-risk group compared to the low-risk group (**Figures 5E** **, F**). The ROC curve was used to investigate the predictive ability of the Riskscore for patient prognosis and the area under the curve (AUC) values for 1-, 2-, 3-years were calculated. In the training cohort, the AUC values for determining the accuracy of Riskscore as a predictive marker for 1-, 2-, 3-years were 0.794, 0.849, and 0.879, respectively (**Figure 5G**). Further, in the validation cohort, the AUC values for 1-, 2-, and 3-years were 0.622, 0.679, and 0.723, respectively (**Figure 5H**). Based on these results, the established risk model had the potential to predict the prognosis of IPF patients.

**Figure 6:**
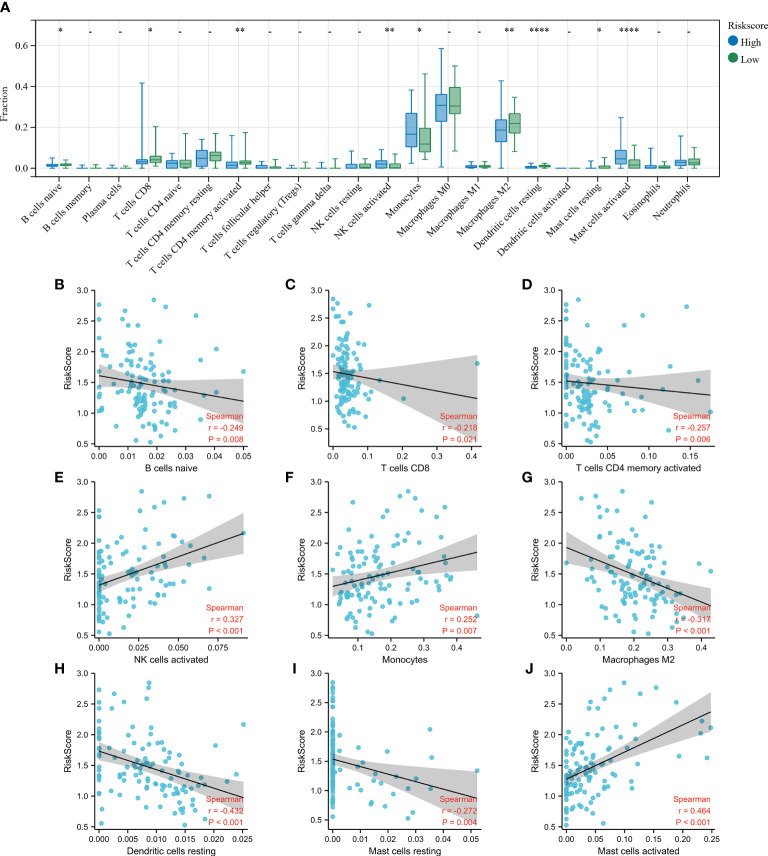
Estimation of immune cell infiltration in the bronchoalveolar lavage fluid (BALF). **(A)** Level of infiltration in the high- and low- Riskscore groups using the CIBERSORT algorithm. The plots show the differences in immune infiltration scores between the high- and low- Riskscore groups. **(B–J)** The Spearman’s rank correlation between Riskscore and the fraction of BALF immune cells is shown. Scatterplots show that the Riskscore negatively correlated with naive B cells **(B)**, CD8 T cells **(C)**, activated memory CD4 T cells **(D)**, M2 macrophages **(G)**, resting dendritic cells **(H)**, and resting mast cells **(I)**, while Riskscore positively correlated with activated NK cells **(E)**, monocytes **(F)**, and activated mast cells **(J)**. *p < 0.05; **p < 0.01; and ****p < 0.0001.

Finally, the CIBERSORT algorithm was used to determine the differences in infiltration levels of different immune cells between the two groups and the association between the Riskscore and the immune cell infiltration in the BALF (**Figure 6A**). Patients with elevated Riskscore showed increased levels of activated mast cells, monocytes, and activated NK cells, and the levels of resting mast cells, naive B cells, resting dendritic cells, M2 macrophages, activated memory CD4, and CD8 T cells were relatively lower. Correlation analysis reveal an inversely correlation between Riskscore and the naive B cells (**Figure 6B**; r = −0.249, p < 0.01), CD8 T cells (**Figure 6C**; r = −0.218, p < 0.05), activated memory CD4 T cells (**Figure 6D**; r = −0.257, p < 0.01), M2 macrophages (**Figure 6G**; r = −0.317, p < 0.001), resting dendritic cells (**Figure 6H**; r = −0.432, p < 0.001), resting mast cells (**Figure 6I**; r = −0.272, p = 0.004). A positive correlation was observed between activated NK cells (**Figure 6E**; r = 0.327, p < 0.001), monocytes (**Figure 6F**; r = 0.252, p < 0.01), activated mast cells (**Figure 6J**; r = 0.464, p < 0.001) and Riskscore. These results show a close correlation between the constructed risk model and the level of immune cell infiltration in the BALF of IPF patients.

### Established risk model’s independence

**Figure 7:**
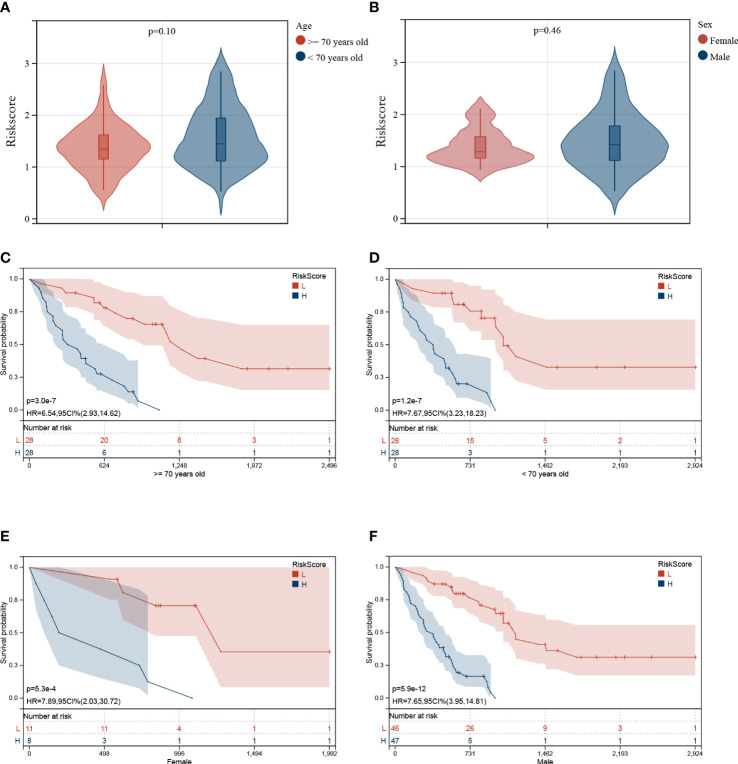
Association between Riskscore and clinical characteristics. No significant difference was observed in patients of different ages **(A)** and sex **(B)**. Independence analysis of the risk model **(C–F)**. The survival curve of IPF patients regrouped based on age **(C, D)** and sex **(E, F)**.

Furthermore, the correlation between the Riskscore and clinical characteristics was evaluated, and the independence of the risk model was verified by performing subgroup and regression analysis. There was no difference in the Riskscore between IPF patients of different ages (**Figure 7A**) and sex (**Figure 7B**), suggesting no correlation between Riskscore and clinical parameters. Once the patients were classified based on age (**Figures 7C** **, D**) and sex (**Figures 7E** **, F**), the risk model demonstrated powerful predictive ability. The IPF patients with lower Riskscore had more favorable outcomes. Additionally, univariate and multivariate Cox regression analysis show that the developed risk model could independently serve as a predictive indicator for the prognosis of IPF patients (**Table 2**). These results suggest that the constructed risk model demonstrated independence in predicting the prognosis of IPF patients.

**Table 2:** Univariate and multivariate analysis of Riskscore and characteristics in the training cohort.

Variates	Total (n)	Univariate analysis		Multivariate analysis	
		Hazard ratio (95% CI)	P-value	Hazard ratio (95% CI)	P-value
Age	112	0.986 (0.963-1.010)	0.255		
Sex	112				
Male	93	Reference			
Female	19	0.810 (0.436-1.504)	0.505		
Riskscore	112	10.354 (5.919-18.113)	<0.001	10.354 (5.919-18.113)	<0.001

CI, Confidence interval.

### Development of a predictive nomogram

**Figure 8:**
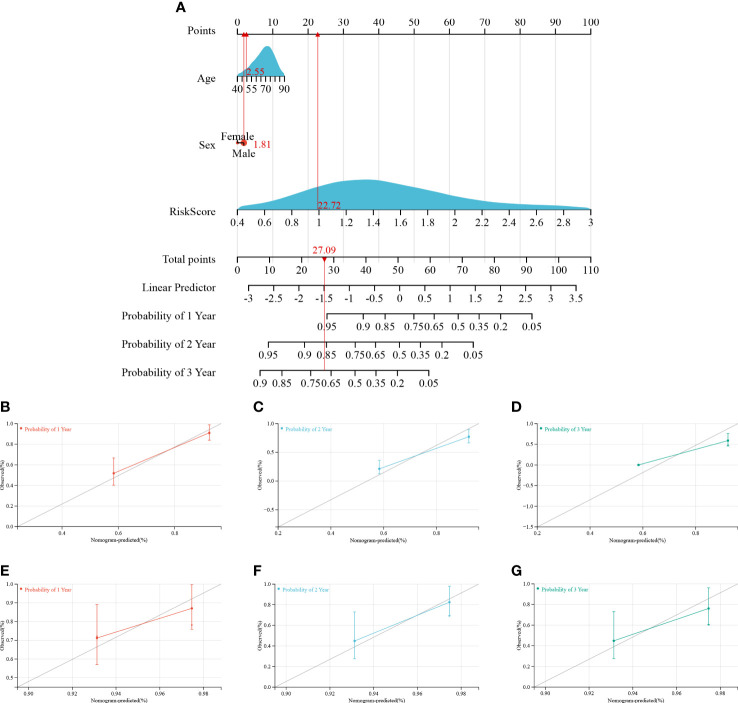
Construction and calibration of the nomogram. **(A)** Nomogram integrating risk score and clinical features, **(B–D)** calibration of the nomogram at 1-, 2-, and 3- years in the training cohort, and **(E–G)** calibration of the nomogram at 1-, 2-, and 3- years in the verification cohort.

A predictive nomogram was constructed to estimate the prognosis risk of IPF patients by calculating the chances of survival of patients 1, 2, and 3 years after diagnosis (**Figure 8A**). The accuracy of the nomogram was verified by testing it on training and validation cohorts. The C-index, the calibration curve **(** **Figures 8B** **–G** **)**, and decision curve analysis **(** **Figure S2** **)** demonstrated satisfactory accuracy of the diagnostic nomogram model. In the training cohort, the C-index for the nomogram was 0.7642 (95% CI: 0.7125-0.7962), and the C-index for the validation cohort was 0.6906 (95% CI: 0.5578-0.8234). The actual overall survival of the patients for 1, 2, and 3 years was very similar to the predicted overall survival of the patients in the training cohort **(** **Figures 8B** **–D** **)**. Consistent results were observed in the validation cohort **(** **Figures 8E** **–G** **)**. Based on these results, it is tempting to conclude that the nomogram could accurately predict the prognosis of IPF patients.

## Discussion

The condition of IPF patients worsens over time and could be potentially fatal. Very few therapeutic options are available for the treatment of IPF patients, and the therapeutic outcomes are poor and have an unfavorable prognosis. Despite the advancements in diagnosis and therapeutic strategies, the survival rate in IPF patients has not improved. The clinical progression of IPF is highly variable and unpredictable; hence, better risk assessment strategies and personalized, targeted therapy for IPF patients are necessary (32).

In this study, two subgroups of IPF patients were identified based on the expression profiles of genes associated with FA metabolism and had significantly different overall survival. The enrichment analysis revealed that the two subgroups enriched different immune and metabolism pathways. Further, the immune analysis revealed that two subgroups had different immune cell infiltration patterns. Additionally, a predictive risk model based on FAMRGs was developed and could accurately predict the prognosis of IPF patients. Therefore, the results of our study shed light on developing IPF-specific therapies.

Consensus clustering was used to classify the IPF patients into different groups based on the gene expression matrix, and the results were reliable. Two subgroups were initially identified by consensus clustering based on the FAMRGs expression in IPF patients. The results revealed that the two subgroups had significantly different overall survival. These findings further confirmed that the FA metabolic subtypes in IPF patients influence the prognosis, and the prediction models constructed based on FAMRGs were reliable.

Furthermore, the enrichment analysis was conducted between the two subgroups to investigate the inherent biological processes. GSEA was used to elucidate the gene expression pattern in various subgroups (33). GSEA results revealed the enrichment of immune-related pathways, including natural killer cell-mediated cytotoxicity, B cell receptor signaling pathway, Toll-like receptor signaling pathway, leukocyte transendothelial migration, chemokine signaling pathway, and Nod-like receptor signaling pathway in cluster 1 with poor outcomes. Based on the results, it is likely that dysregulated immune system could predict the role of FA metabolism in the onset and progression of IPF. Further, the association between FA metabolism and immune dysregulation could be regulated by an epigenetic process such as DNA methylation influenced by genetic variation (34). The results showed that cluster 1 and cluster 2 had different metabolic signatures. The pathways enriched in cluster 1 were predominantly linked to the metabolism of amino acids, including arginine, proline, and histidine metabolism, and lipid metabolism pathways, including the metabolism of glycerophospholipid and arachidonic acid. Short-chain FA metabolism pathways, including propanoate metabolism and butanoate metabolism, and other metabolism pathways, including aminoacyl tRNA biosynthesis, riboflavin metabolism, limonene, and pinene degradation, were mainly enriched in cluster 2. Since the classification was based on FA metabolism-related genes, the result showed that the crosstalk between FA metabolism and other nutrient metabolism could affect IPF pathogenies and was worth further exploration.

As mentioned previously, immune systems play an integral role in the onset and progression of IPF and are also associated with FA metabolism. The CIBERSORT algorithm is a tool that analyses biological information and evaluates the expression levels of immune cells based on RNA-seq data, and obtains ratios of different immune cells from samples. It is extensively used to examine the infiltration of immune cells in various diseases, such as tumors, osteoarthritis, and lupus nephritis (35–37). Li et al. established a hypoxia-immune-based prediction model to determine the prognosis among IPF patients using CIBERSORT (38). Hence, CIBERSORT was used to determine the level of immune cell infiltration in the two clusters. The results revealed the presence of several immune cells in BALF of patients that could potentially be associated with IPF prognosis. The immune cells identified were mainly activated and resting mast cells, naive B cells, M0 and M2 macrophages, monocytes, activated and resting NK cells, and resting dendritic cells. Increased infiltration of activated NK cells, activated mast cells, and monocytes were observed in patients in cluster 1 with poor survival outcomes. Similarly, in the constructed risk model, an increase in infiltration of these three immune cell populations was observed in the high-risk group with poor prognosis and was positively correlated with Riskscore. Previous studies have shown that pulmonary fibrosis patients had higher levels of NK cell infiltration in BALF compared to patients with sarcoidosis (39). Scott et al. indicated that elevated levels of circulating monocyte count could be a cellular biomarker for poor outcomes in IPF patients (40). Kawanami et al. have shown that patients with fibrotic lung disease had significantly higher numbers of mast cells in their lungs. Mast cells are often localized around the thickened regions of the alveolar septa and are located near abnormal epithelial cells (41). Taken together, these results revealed that activated mast cells, monocytes, and activated NK cells in the BALF of patients with IPF may promote disease progression.

Based on our results, it is tempting to infer that dysregulation in FA metabolism results in the disturbance in the immune system and metabolism in BALF of IPF patients, thereby contributing to the poor prognosis of the IPF patients. As aforementioned, reprogramming FA metabolism was identified as a distinctive characteristic in IPF patients with poor prognoses. To further verify the influence of FA metabolic disorders on IPF and examine the prognostic value of FAMRGs in IPF patients, a prognostic risk model using FAMRGs was created and tested on a separate validation cohort. The results showed that five genes used to establish the risk model were remarkably associated with the onset and progression of IPF. Gamma-glutamyltransferase 5 (GGT5) cleaves glutathione peptides to maintain the glutathione balance in the human body (42). Previous research has shown that mice lacking gamma-glutamyl transpeptidase developed a less severe bleomycin-induced pulmonary fibrosis (43). However, the role of GGT5 in FA metabolism and the progression of IPF is unknown. Branched-chain acyl-CoA oxidase (*ACOX2)*, a peroxisomal enzyme, plays a role in the metabolism of bile acid intermediates and branched-chain FAs. Further, *ACOX2* deficiency has been linked to an increased risk of developing liver fibrosis (44), but its role in pulmonary fibrosis is unknown. Leukotriene-B4 omega-hydroxylase (*CYP4F3)* consists of enzymes CYP4F3A and CYP4F3B, responsible for the metabolism of leukotriene B4 and 5-hydroxyeicosatetraenoic acid *via* omega oxidation reaction, which results in the inhibition and deterioration of inflammatory markers  (45, 46). *CYP4F3* is associated with inflammatory diseases, like inflammatory bowel disease (47); however, the role of CYP4F3 in IPF is unknown. 3-hydroxyacyl-CoA dehydratase 4 (HACD4) is involved in elongation, and the biosynthesis of very-long-chain FA; therefore, its role in IPF is worth exploring. Further, *ODC1* encodes for rate-limiting enzymes associated with the polyamine biosynthetic pathways and acts as a catalyst for converting ornithine to putrescine. This indicates the significance of *ODC1* in IPF. The survival analysis demonstrated that the developed risk model had an excellent ability to predict the survival of IPF patients in both cohorts. The prognosis of the IPF patients could be determined independently by each of the five genes. The independent and subgroup analysis demonstrated that the FAMRGs-based risk model could independently predict the prognosis of IPF patients, regardless of age and sex. Ultimately, a nomogram incorporating the Riskscore and the clinical characteristics was developed, calibrated, and tested. The results revealed that it had a powerful ability to predict patient survival. Taken together, these results provide additional evidence that FAMRGs have a prognostic and predictive role in IPF.

Due to the poor prognosis, high variability, and unpredictable nature of IPF progression, there is a need for efficient risk classification and treatment strategy for developing personalized, targeted therapy. Our results demonstrate highlights of FA metabolism in comparison to earlier studies. Our study was based on FA metabolism, which has gained considerable attention in IPF-related research. Using consensus clustering, two molecular clusters with different patient prognoses and immunological statuses were identified. Second, the biological processes based on consensus clustering results were identified, and the fundamental mechanisms associated with them were partially elucidated. Lastly, the role of FA metabolism on the infiltration of immune cells in BALF of IPF patients was elucidated. However, the role of five selected genes in IPF is unclear, and further studies are necessary to understand their role in IPF. Our results offer a good theoretical foundation for research on IPF.

However, our study also has several limitations. First, we could not establish the involvement of FAMRG in the progression of IPF due to insufficient data regarding the pulmonary function of IPF patients. Second, our results were based on bioinformatics analysis, and hence further experimental validation is required. Thirdly, the data used for the study were retrieved from publicly available databases since our clinical practice had an insufficient number of IPF patients. Therefore, further studies evaluating the performance of the prognostic model and the mechanism of the five FAMRGs in the IPF pathogenesis using molecular biology tools and prospective cohort studies are required.

## Conclusions

In this study, consensus clustering was used to identify two distinct molecular clusters based on the FAMRGs in IPF patients. The enrichment and immune analyses show that dysregulation of FA metabolism contributes to disorders of the immune system and nutrition metabolism, which result in poor prognosis. Our study may shed light on developing novel targeted treatment approaches and offer a theoretical basis for personalized treatment strategies.

## Data availability statement

Publicly available datasets were analyzed in this study. This data can be found here: https://www.ncbi.nlm.nih.gov/geo/query/acc.cgi?acc=GSE70866.

## Author contributions

HZ and YL designed the study and revised the manuscript. YL and CG collected the data. YL analyzed and interpreted the data and drafted the manuscript. All authors have read and approved the final manuscript.

## Funding

This study was supported by grants from the Social Development Projects of Key R&D Programs in Jiangsu Province (BE2019643), the National Natural Science Foundation of Jiangsu Province (BK20171178), General Program of Jiangsu Commission of Health (H2017083), and the Project of Invigorating Health Care through Science, Technology, and Education, Jiangsu Provincial Medical Youth Talent (QNRC2016778).

## Conflict of interest

The authors declare that the research was conducted in the absence of any commercial or financial relationships that could be construed as a potential conflict of interest.

## Publisher’s note

All claims expressed in this article are solely those of the authors and do not necessarily represent those of their affiliated organizations, or those of the publisher, the editors and the reviewers. Any product that may be evaluated in this article, or claim that may be made by its manufacturer, is not guaranteed or endorsed by the publisher.
