# Supplementary material for: Fatty acid metabolism-related genes in bronchoalveolar lavage fluid unveil prognostic and immune infiltration in idiopathic pulmonary fibrosis
Source: Front Endocrinol (Lausanne). 2022 Oct 4;13:1001563. doi: 10.3389/fendo.2022.1001563 (PMC9576944; doi:10.3389/fendo.2022.1001563)
Supplement: Supplementary file 1 [file DataSheet_1.docx]

**Table S1.** Significant genes generated in Univariate Cox regression analysis.

| **Genes** | **HR** | **HR 95%CI**  **(Lower)** | **HR 95%CI**  **(Upper)** | **P-value** |
| --- | --- | --- | --- | --- |
| ACOX2 | 1.913156645 | 1.587611125 | 2.305456475 | 9.47E-12 |
| HACD4 | 0.162350702 | 0.085267208 | 0.309119424 | 4.14E-08 |
| ALDH2 | 0.480671654 | 0.366963379 | 0.629613886 | 5.08E-08 |
| CYP4F3 | 1.320493389 | 1.179013045 | 1.478951228 | 2.83E-07 |
| GGT5 | 1.809798917 | 1.427763175 | 2.29405841 | 2.03E-06 |
| ACOT11 | 2.343499107 | 1.630017028 | 3.369282635 | 9.90E-06 |
| HPGDS | 0.600289885 | 0.478455566 | 0.753148195 | 1.09E-05 |
| ODC1 | 5.872295932 | 2.6627765 | 12.95033943 | 1.46E-05 |
| G0S2 | 1.378016917 | 1.188671808 | 1.597523059 | 1.77E-05 |
| ACSM3 | 0.605830774 | 0.480312976 | 0.764149513 | 2.36E-05 |
| PTS | 4.645489159 | 2.17730339 | 9.911604248 | 7.61E-05 |
| PECR | 0.490169387 | 0.34343367 | 0.699599512 | 7.97E-05 |
| SLC27A3 | 0.405982875 | 0.259144001 | 0.636025123 | 8.92E-05 |
| REEP6 | 0.436625613 | 0.284678782 | 0.669673814 | 0.000138066 |
| AOC3 | 0.744360989 | 0.639158559 | 0.866879234 | 0.000157593 |
| DPEP2 | 0.350896039 | 0.202631951 | 0.60764371 | 0.000182109 |
| CYP4F2 | 1.66146946 | 1.242343368 | 2.221995013 | 0.000259273 |
| OLAH | 1.238869748 | 1.101938898 | 1.392816114 | 0.000356092 |
| CYP2J2 | 1.543032978 | 1.216720096 | 1.956859905 | 0.000364054 |
| SCD | 0.766953357 | 0.656775907 | 0.895613626 | 0.00044101 |
| PON3 | 1.473861011 | 1.184004806 | 1.834676912 | 0.000489788 |
| HSD17B8 | 0.408368594 | 0.254788052 | 0.654524052 | 0.000522598 |
| HSD17B11 | 0.409475738 | 0.247251792 | 0.678136157 | 0.00057918 |
| MAOA | 1.409336888 | 1.158216138 | 1.714904844 | 0.000630087 |
| PTGDS | 1.388505947 | 1.148731425 | 1.678328565 | 0.000747646 |
| YWHAH | 0.464868127 | 0.298019231 | 0.725128961 | 0.00082167 |
| CYP4B1 | 1.330049628 | 1.12399153 | 1.573883758 | 0.000830059 |
| CYP2U1 | 0.625253185 | 0.47109165 | 0.829863032 | 0.001092994 |
| ACOXL | 1.377743847 | 1.137524962 | 1.668691387 | 0.001119182 |
| ACSBG1 | 1.35298098 | 1.121012595 | 1.632950013 | 0.001462962 |
| UGDH | 1.317021668 | 1.121959599 | 1.545996912 | 0.001547867 |
| MECR | 0.377171455 | 0.206054268 | 0.690392428 | 0.001614027 |
| HSD17B7 | 0.471748267 | 0.293218318 | 0.758978597 | 0.002023396 |
| SCP2 | 0.498689165 | 0.319241716 | 0.779004969 | 0.002190267 |
| FADS2 | 0.543153029 | 0.365081906 | 0.808079524 | 0.002554902 |
| NDUFAB1 | 4.599758156 | 1.667645921 | 12.68721065 | 0.003033431 |
| FMO1 | 0.711245101 | 0.568513355 | 0.889811276 | 0.003181626 |
| GPD1 | 0.77826985 | 0.659972935 | 0.917770908 | 0.003268555 |
| MIF | 3.307041045 | 1.460000125 | 7.490766804 | 0.004148691 |
| ACOT4 | 0.661040045 | 0.494960009 | 0.882846964 | 0.004382797 |
| THEM5 | 1.172210293 | 1.048559587 | 1.310442428 | 0.004473948 |
| CBR3 | 1.586989384 | 1.155048727 | 2.180458058 | 0.005451959 |
| ECH1 | 0.400283189 | 0.210494252 | 0.761192431 | 0.005517282 |
| PTGS2 | 1.373663498 | 1.098582567 | 1.717623657 | 0.005666988 |
| ACSL1 | 1.806601608 | 1.182256878 | 2.760660082 | 0.006417458 |
| FAAH2 | 1.279865075 | 1.069251887 | 1.531963264 | 0.006618661 |
| PRXL2B | 1.758746641 | 1.174196911 | 2.634302407 | 0.006655812 |
| HADH | 0.45392445 | 0.257286944 | 0.800846724 | 0.006774219 |
| GPD2 | 0.58741456 | 0.399588544 | 0.863527924 | 0.007269941 |
| CA4 | 1.165328094 | 1.040108698 | 1.305622739 | 0.00742026 |
| SLC27A2 | 1.261434423 | 1.062500005 | 1.497615808 | 0.007729538 |
| LDHA | 3.412729666 | 1.368230499 | 8.51225271 | 0.00844135 |
| DECR1 | 0.459962525 | 0.258136038 | 0.819589262 | 0.008549358 |
| ACOX1 | 0.481308796 | 0.278032758 | 0.833204543 | 0.009085227 |
| CYP1B1 | 1.177058682 | 1.037575525 | 1.335292812 | 0.01098294 |
| PTGS1 | 1.563444336 | 1.106974849 | 2.208142482 | 0.011324116 |
| LTA4H | 0.510070557 | 0.301969976 | 0.861582258 | 0.011716763 |
| ALOX15B | 1.254822417 | 1.051696448 | 1.497180389 | 0.011828247 |
| FADS1 | 0.643821709 | 0.455044963 | 0.910913046 | 0.012829943 |
| PTPRG | 1.331856286 | 1.062473815 | 1.669538713 | 0.013278898 |
| IL4I1 | 1.420397295 | 1.073563198 | 1.879282449 | 0.014027541 |
| VNN1 | 1.232247026 | 1.042084909 | 1.457110376 | 0.014348185 |
| PON1 | 0.539185863 | 0.328292418 | 0.885556225 | 0.01469825 |
| DBI | 3.140951894 | 1.25149377 | 7.88304268 | 0.015180041 |
| ALOX5 | 0.572705538 | 0.365279458 | 0.897919734 | 0.015434716 |
| GPX2 | 1.494702991 | 1.089688388 | 2.050253133 | 0.015665636 |
| ACSF2 | 0.674922316 | 0.505239636 | 0.901592235 | 0.016219553 |
| ACOT1 | 0.686691501 | 0.506254518 | 0.931439031 | 0.016462825 |
| CD36 | 1.369691943 | 1.055286656 | 1.777769109 | 0.017861185 |
| LGALS1 | 1.986985362 | 1.124680027 | 3.510430285 | 0.01804035 |
| IDH1 | 2.372640329 | 1.180940649 | 4.766896741 | 0.018123097 |
| ADIPOR2 | 0.522812334 | 0.303618279 | 0.900251256 | 0.019419523 |
| HSD17B4 | 1.931221607 | 1.092502497 | 3.41382917 | 0.024788898 |
| ACOT7 | 0.698897516 | 0.508813068 | 0.95999448 | 0.027183442 |
| ALDH1A1 | 0.706347853 | 0.524527077 | 0.951194537 | 0.027461475 |
| MCAT | 0.49963638 | 0.270327553 | 0.923459372 | 0.028064485 |
| SLC25A1 | 0.569074814 | 0.344257508 | 0.940709022 | 0.028243818 |
| SLC25A20 | 0.48264905 | 0.251165287 | 0.927477313 | 0.029229382 |
| AMACR | 0.595850514 | 0.374297582 | 0.948544291 | 0.029331376 |
| AADAT | 2.601741721 | 1.091438619 | 6.201961215 | 0.02966954 |
| TECR | 0.525305579 | 0.294366842 | 0.937421993 | 0.029781235 |
| CA2 | 0.78997167 | 0.63768822 | 0.978621245 | 0.03119443 |
| HSD17B10 | 0.445792852 | 0.213704857 | 0.929933316 | 0.03119799 |
| ALDH3A2 | 0.623263508 | 0.412273708 | 0.942231805 | 0.031449616 |
| PDHB | 1.835115492 | 1.04053252 | 3.236466715 | 0.035685672 |
| BLVRA | 0.451810693 | 0.215047432 | 0.949245943 | 0.036812415 |
| CPT2 | 0.550946 | 0.319532803 | 0.949954095 | 0.036881814 |
| HACL1 | 0.493123587 | 0.25353185 | 0.959133425 | 0.036893302 |
| DHCR24 | 0.739521753 | 0.557074895 | 0.98172154 | 0.037493374 |
| CIDEA | 1.224991129 | 1.008440187 | 1.488043897 | 0.04050894 |
| FABP2 | 1.177057741 | 0.998186131 | 1.387982543 | 0.04349257 |
| ENO3 | 1.671470288 | 1.014142723 | 2.75485182 | 0.045255791 |
| INMT | 0.819164773 | 0.674504815 | 0.994849719 | 0.046109976 |
| BMPR1B | 1.375690861 | 1.001214625 | 1.890229425 | 0.046556797 |
| THRSP | 1.161765758 | 0.998901934 | 1.351183365 | 0.049919097 |

HR, hazard ratio; CI, confidence interval.


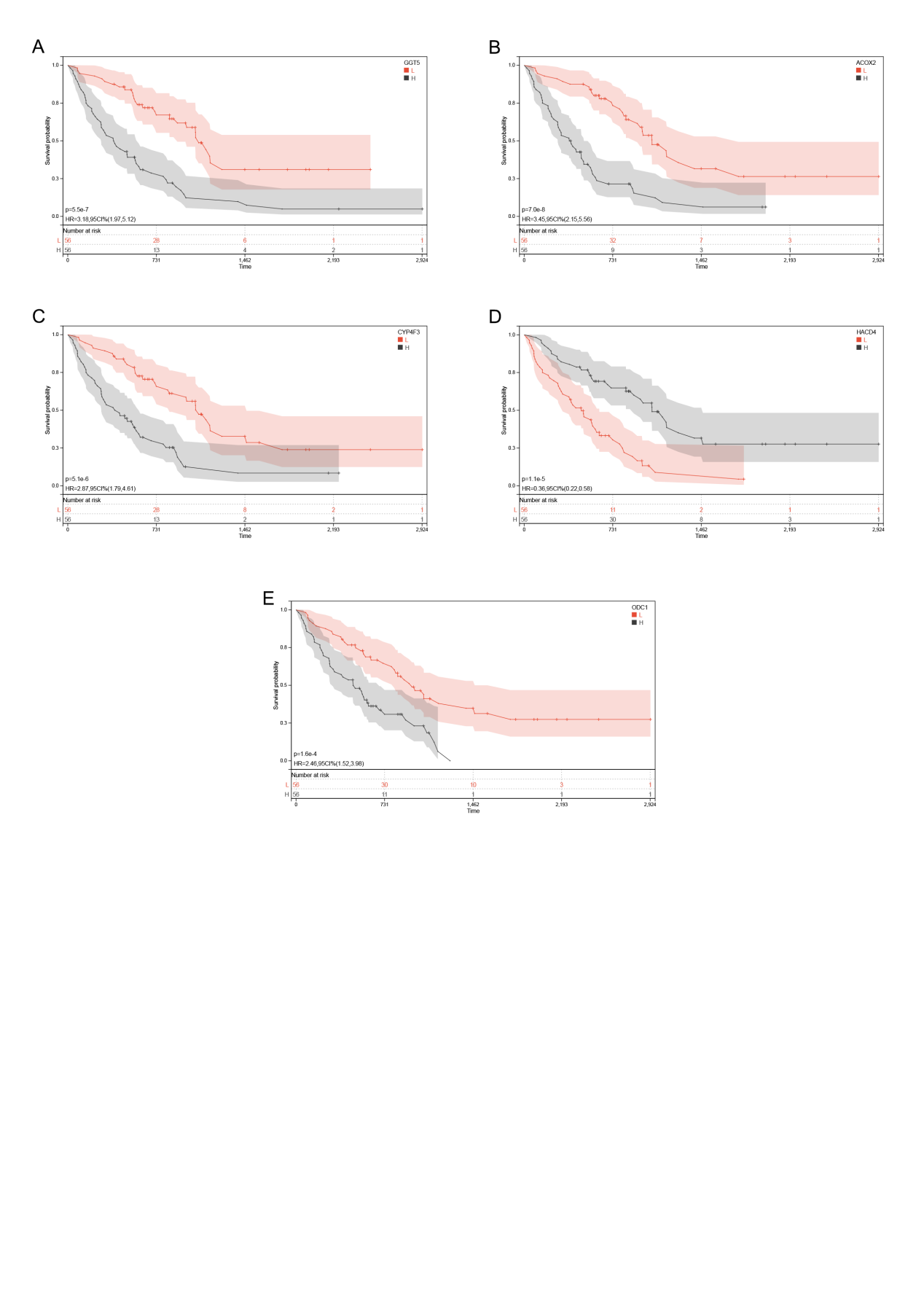


**Figure S1.** Survival analysis of the five candidate genes. All of GGT5, ACOX2, CYP4F3, HACD4 and ODC1 were independently prognostic gene in IPF.


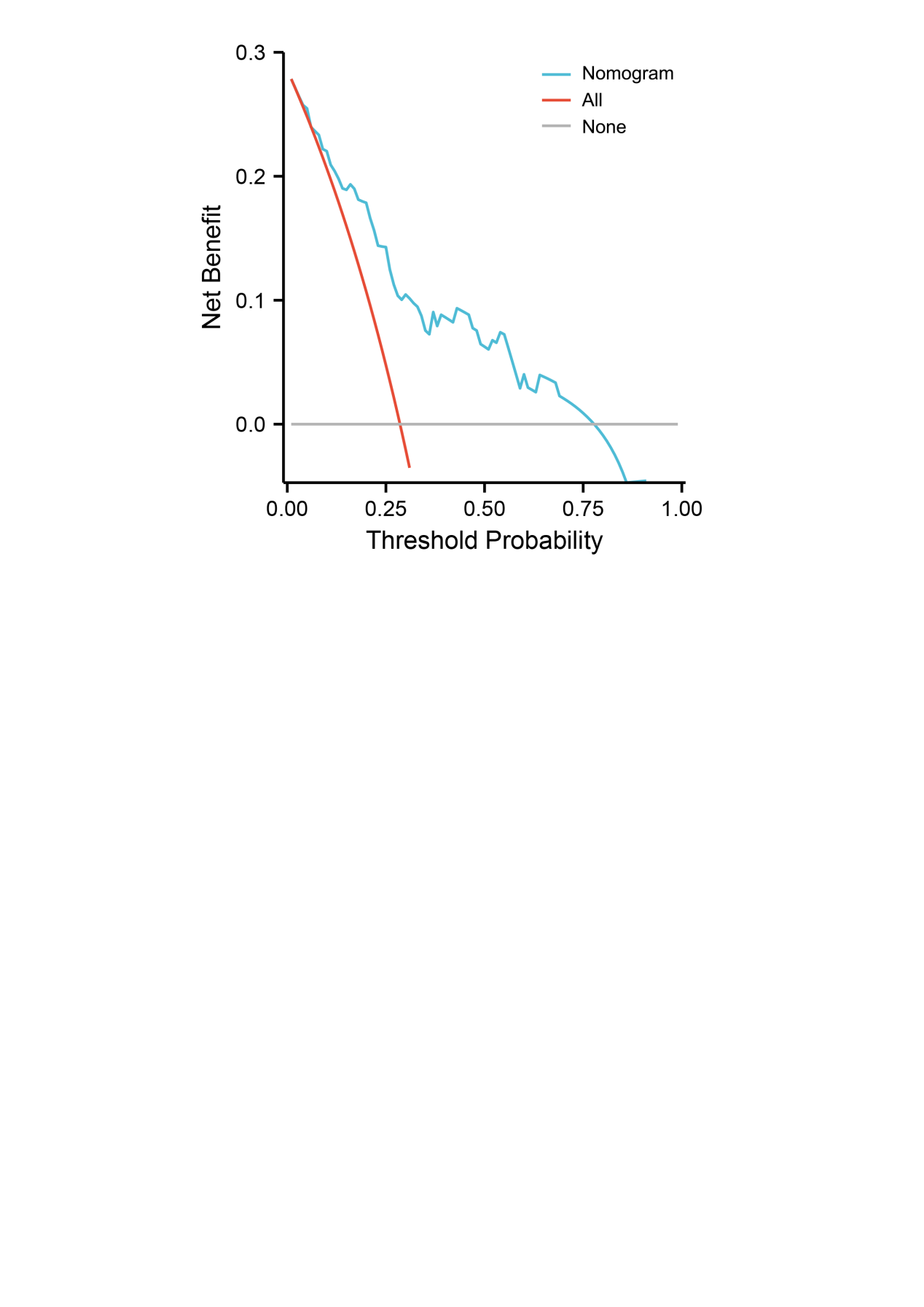


**Figure S2.** Decision curve Analysis of the nomogram.
